# The effect of social media use on employment anxiety of college students: the mediating role of social support

**DOI:** 10.3389/fpsyg.2025.1477306

**Published:** 2025-09-11

**Authors:** Feng Li, Liangkun Chen, Lexin Huang, Suwei Ma

**Affiliations:** ^1^School of Marxism, Jiangnan University and School of Marxism, Wuxi University of Technology, Wuxi, China; ^2^School of Marxism, Jiangnan University, Wuxi, China; ^3^School of Philosophy and Social Development, South China Normal University, Guangzhou, China

**Keywords:** social media use, employment anxiety, social support, structural equation modeling, college students

## Abstract

Under the dual context of labor market transformations and the widespread adoption of social media, college students face increasingly severe employment pressure. This study integrates social comparison theory and stress coping theory to systematically explore the complex relationships among social media use, social support, and employment anxiety through structural equation modeling (SEM) analysis of questionnaire data from 400 Chinese college students. The findings reveal that: (1) High-frequency social media use is significantly positively correlated with employment anxiety, with mechanisms involving the depletion of psychological resources due to information overload and passive social comparison; (2) Social support exhibits a paradoxical mediating role: while online support is strongly associated with social media use, its indirect effects suggest that online interactions may exacerbate anxiety through irrational competition and superficial emotional feedback. This phenomenon is termed the “reinforcement paradox” of online support—a paradoxical mechanism where digital socialization, intended to alleviate stress through peer connection, instead amplifies anxiety by creating self-reinforcing cycles of social comparison and emotional dependency. This paradox arises from the dual-edged nature of online interactions: while providing perceived support, they simultaneously normalize competitive benchmarks and reduce emotional feedback to performative gestures, which collectively heighten psychological strain;(3) Significant gender and grade differences exist, with female participants exhibiting significantly higher anxiety levels than males, and upperclassmen showing escalating anxiety as job-seeking deadlines approach. This study is the first to uncover the theoretical framework of the “reinforcement paradox” in digital social support systems, providing a breakthrough in understanding how virtual networks simultaneously buffer and exacerbate mental health challenges. On a practical level, it is recommended to establish a collaborative online-offline support system and optimize social media information filtering mechanisms to alleviate anxiety. Future research should expand cross-cultural comparisons and incorporate variables such as psychological resilience and self-efficacy to further refine intervention frameworks.

## 1 Introduction

Changes in domestic and international market conditions, coupled with economic structural adjustments, have exerted a severe impact on China’s labor market. The nation’s rapid economic transformation has led to shifts in industry demands, creating a mismatch between available jobs and the skills of job seekers. Additionally, the popularization of higher education has led to a large number of college graduates entering the labor market, thereby increasing employment pressure. According to data from the Ministry of Education of China, the scale of college graduates in the class of 2024 is expected to reach 11.79 million, an increase of 210,000 year-on-year (Xinhua, 2024). This surge in graduates intensifies competition for limited job opportunities, exacerbating employment anxiety among young job seekers. Employment anxiety among college students is becoming more pronounced due to these factors. The uncertainty of securing suitable employment in a competitive market can lead to stress, anxiety, and even depression among graduates. Simultaneously, as social media becomes increasingly popularized and widely used among college students, it plays a significant role in their daily lives. A survey conducted by *China Youth Daily and China Youth Campus Media* in 2022 revealed that 99.39% of college students use social media in their daily lives (Ma and Fan, 2024). Within China’s unique social media ecosystem—marked by the high penetration and functional integration of platforms such as WeChat, Weibo, Xiaohongshu (Red), and TikTok—social media has become a critical platform for college students to access information, exchange ideas, and express emotions (Oh and Syn, 2015). Young people increasingly prefer to seek support online, leveraging social media to connect with peers, mentors, and potential employers.

Young individuals show a greater inclination to seek support online, utilizing social media to connect with peers, mentors, and potential employers. This online interaction can provide emotional support and practical advice, which may help alleviate some of the stress associated with job hunting (Horgan and Sweeney, 2010). However, the constant exposure to the successes and failures of others on social media can also lead to increased self-comparison and pressure, contributing to feelings of inadequacy and anxiety. To understand the impact of social media use on employment anxiety, key socio-psychological theoretical frameworks are essential. First, Social Comparison Theory (Festinger, 1954) provides a foundational perspective: social media platforms are saturated with others’ job-hunting successes and curated life displays, which easily trigger upward social comparisons among college students. This phenomenon, combined with the observational learning mechanism emphasized by Social Cognitive Theory (Bandura, 1986), may lead individuals to internalize unrealistic standards of success, intensifying self-doubt and stress (i.e., the more “success stories” one observes, the more inadequate one feels). Stress and Coping Theory (Lazarus and Folkman, 1984) further explains how students navigate these amplified stressors—particularly within China’s highly interactive, relationship-driven social media environment. Social media itself may serve as a channel for seeking information or emotional release (active coping), but it can also become a stressor itself due to information overload, exposure to negative content, and persistent upward comparisons, leading to maladaptive responses (e.g., avoidance, excessive worry). Within this complex stress-coping dynamic, Social Support Theory (Cohen and Wills, 1985) highlights how emotional, informational, and instrumental support from both online and offline sources might buffer the pressures of social media use or mediate its relationship with employment anxiety. However, in China’s unique social media cultural context—dominated by close-contact networks like WeChat Moments, interest- or career-based platforms like Xiaohongshu (Red) and Maimai, and short-video platforms like TikTok—these theoretical mechanisms may manifest in specific ways. For instance, support within strong-tie networks (e.g., WeChat) may be more direct but also carry higher comparative pressure, while weak-tie networks (e.g., Maimai, Xiaohongshu) may offer broader information access but with limited emotional depth. Thus, investigating how social media use influences employment anxiety through perceived and accessible social support quality in the Chinese context holds significant theoretical and practical implications.

In this context, exploring the relationship between college students’ employment anxiety and social media use, as well as the mediating role of social support, is of great significance for alleviating the employment pressure on college students and promoting the healthy growth of young people. Understanding how social media can be both a source of stress and a tool for support will help in developing strategies to better manage employment anxiety among graduates. This could involve promoting positive online interactions, providing mental health resources, and fostering environments where students feel supported both online and offline.

### 1.1 Employment anxiety: core concept and influencing factors

Employment anxiety refers to the psychological state of worry, tension, and unease experienced by individuals under job-seeking pressure (Zhao, 2023). Existing research generally treats it as an outcome variable, exploring its influencing factors and intervention pathways. Studies indicate that employment anxiety not only harms individual mental health and career development but also affects social harmony and stability (Unguren and Huseyinli, 2020). Its causes can be categorized as external and internal factors: Among external factors, social support has been proven to be a key protective factor significantly suppressing anxiety levels (Jiaxin, 2023). Internal factors primarily involve psychological capital, with individuals possessing high psychological capital more likely to adopt proactive coping strategies to reduce anxiety (Luthans and Broad, 2022; Yang et al., 2020). Environmental factors (e.g., labor market pressure) also warrant attention (Chen and Zeng, 2021).

The popularity of social media (SM) profoundly impacts university students’ emotional states. Substantial evidence shows that excessive or problematic social media use (PSMU) is significantly associated with heightened anxiety levels (Dhir et al., 2018; Teng et al., 2022; Li et al., 2024; Bhiri and Mapfunde, 2025). Its underlying mechanisms include:

Fear of Missing Out (FoMO): Anxiety triggered by fearing missing important information or social events, linked to attention deficits, sleep disorders, and declining academic performance (Gupta and Sharma, 2021; Tandon et al., 2021; Chi et al., 2022). Reducing social media use effectively alleviates FoMO and related anxiety symptoms (Davis and Goldfield, 2024).

Passive Use and Social Comparison: Passive browsing (e.g., viewing others’ curated success stories in job hunting) easily weakens perceived social connection and intensifies self-doubt and anxiety through upward social comparison (Lopes et al., 2022; Wu et al., 2024), particularly regarding appearance anxiety (Wu et al., 2024).

Impaired Real-Life Coping Ability: Over-engagement may diminish individuals’ capacity to handle real-life stressors, divert attention, and erode offline support networks (Li et al., 2024).

Notably, the anxiety impact differs significantly across usage patterns (active vs. passive) (Lopes et al., 2022), and social anxiety often forms a vicious cycle with PSMU (Wu et al., 2024).

Social support plays a central role in mitigating stress and promoting mental health, especially in contexts like academic pressure (Fu, 2024; Huang et al., 2024; Pontes et al., 2024), learning burnout (Qanbary Joopish et al., 2024), and negative emotions (Acoba, 2024; An et al., 2024). Family support typically demonstrates the strongest protective effect (Lin et al., 2024; Yan et al., 2024). Crucially, social support is often regarded as a key mediating mechanism for understanding how social media use affects mental health. For example, it buffers the negative effects of social media dependence on life satisfaction and depressive symptoms (Çi̇çek et al., 2024).

### 1.2 Social media and social support: dynamic and context-dependent relationship

Social media itself serves as a significant channel for obtaining social support. Active use enhances perceived online network responsiveness, boosts perceived social support, alleviates loneliness, and improves life satisfaction (Yue et al., 2024). Online support networks prove particularly vital for specific groups or during special periods (Ibrahim et al., 2024), even moderating social media addiction induced by interpersonal fears (Casale et al., 2024) and alleviating loneliness through enhanced self-efficacy (Jia et al., 2024).

However, the quality and effectiveness of social media-facilitated support exhibit high complexity and duality. Platforms simultaneously deliver supportive and negative/stress-inducing content (Shahiki Tash et al., 2025). The quality of online interactions directly determines their mental health benefits. The relationship between social media use and perceived social support is dynamic and moderated by factors such as social anxiety (Blahošová et al., 2025). Social media can also propagate discriminatory remarks, exacerbating mental health issues among gender minorities (Sánchez-Sánchez et al., 2024). Excessive use without quality feedback may intensify psychological problems (Shahiki Tash et al., 2025).

Particularly within China’s unique social media ecosystem, support acquisition methods, quality perception, and buffering effects may manifest distinct patterns—such as receiving strong relational support alongside high-pressure comparison dynamics, or obtaining algorithm-recommended informational support that is wide-ranging yet shallow in depth.

In summary, existing studies have made certain progress in exploring college students’ employment anxiety, social media use, and social support. However, research focusing specifically on Chinese college students, integrating China’s unique social media ecosystem, and examining the interrelationships among social media use, social support, and employment anxiety remains relatively limited. Therefore, this study aims to address this research gap by empirically investigating the impact of social media use on employment anxiety among Chinese college students within the local context, as well as the mediating role of social support in this relationship. The findings aim to provide culturally adaptive intervention strategies for universities, families, and society to effectively alleviate employment stress and promote the psychological well-being of college students.

### 1.3 Research hypotheses

Based on the existing research findings, this study primarily examines the relationship between college students’ employment anxiety and social media use, as well as the moderating role of social support. Drawing on relevant literature, the following research hypotheses are proposed:

*Hypothesis 1*. There is a positive correlation between young people’s employment anxiety and their use of social media.

*Hypothesis 2*. Social support plays a mediating role between young people’s employment anxiety and their use of social media.

**Figure 1:**
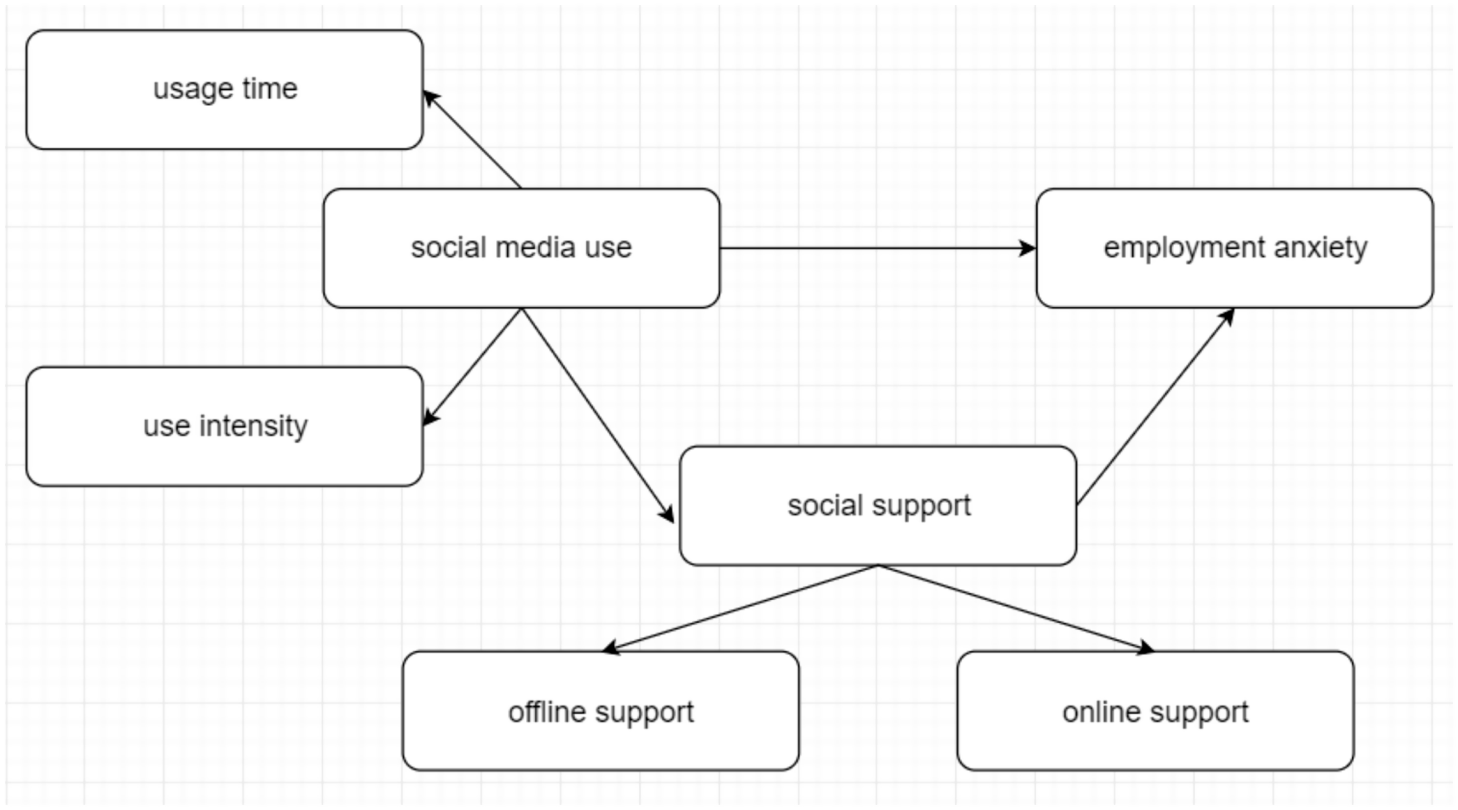
Hypothetical model.

In order to verify the above hypothesis, this study adopts the structural equation modeling (SEM) (Lei and Wu, 2007) with social media use as the independent variable, employment anxiety as the dependent variable, social support as the mediating variable, and gender as the controlling variable. The hypothetical model for this study is shown in Figure 1.

We propose to construct a second-order latent variable mediation model, with social media use as the independent variable, measured through first-order latent variables of usage time and usage intensity. This model posits that social media use influences social support, which in turn affects employment anxiety. The second-order latent variable of social support is measured by its two dimensions: offline support and online support. The model assumes that social media use not only directly influences employment anxiety but also has an indirect effect through social support. First, social media use as an independent variable has been widely found to significantly affect individuals’ psychological well-being. According to social comparison theory (Festinger, 1954), individuals often engage in social comparisons on social media, which can induce anxiety, especially in relation to career development. Seeing others’ professional achievements on social media can lead to self-doubt and increased anxiety. Therefore, the positive relationship between social media use and employment anxiety is theoretically supported (LaRose et al., 2010). Second, social media use is also associated with social support. According to social support theory (Cohen and Wills, 1985), social support plays a crucial role in alleviating psychological stress and anxiety. Social media provides a platform for online interaction, allowing individuals to receive emotional support, informational support, and tangible assistance from others, thus enhancing their social support network. Studies have shown that social media use increases social interactions and can enhance individuals’ sense of social support (Manago et al., 2008). Therefore, the positive relationship between social media use and social support is logically sound. Furthermore, social support is widely considered a key factor in reducing employment anxiety. Numerous empirical studies have shown that strong social support can alleviate emotional burdens, particularly in the context of career uncertainty or stress (Cohen and Wills, 1985). Social support obtained through social media, especially a combination of online and offline support, can effectively reduce employment anxiety. Therefore, the hypothesis that social support mediates the relationship between social media use and employment anxiety is grounded in theoretical and empirical evidence. Finally, we utilize a second-order latent variable model to structure this framework, with social support as a second-order latent variable measured by two first-order latent variables: online support and offline support. This model structure better captures the multidimensional nature of social support, particularly the significant roles of both online and offline support in modern social interactions. Additionally, the use of a second-order latent variable model allows for a clearer understanding of the complex relationships between social media use, social support, and employment anxiety, while testing the significance of the various paths in the model through structural equation modeling (SEM). In conclusion, our second-order latent variable mediation model is theoretically supported by existing research, and it proposes a framework that explores how social media use impacts employment anxiety both directly and indirectly through social support. This model will be tested using Structural Equation Modeling (SEM), which allows for the estimation of relationships between observed and latent variables, providing a robust method for validating the proposed model and its hypotheses.

## 2 Materials and methods

### 2.1 Participants

As mentioned above, the selected subjects of this study are Chinese college students. According to the Chinese government bulletin, the total number of higher education students in China in 2023 will be 47,631,900. In order to generalize from a random sample and avoid sampling error or bias, the random sample needs to be of sufficient size. In this study, the minimum sample size calculation formula was adopted to determine the sample size required for the research (Taherdoost, 2017). Taking the number of Chinese higher education students as the sample size, it was calculated that the minimum sample required was 384 people. The formal investigation of this study uses the questionnaire platform “Wenjuanxing” to issue and collect questionnaires. Through the snowball method, questionnaires were distributed in Wechat group, QQ group and other online social platforms, 420 copies were recovered, and questions with too short answering time and obvious logic errors were eliminated, and 400 valid samples were obtained.50.5% of the participants were female and 49.5% were male. The sample included 27.00% first-year college students, 27.00% second-year students, 21.25% third-year students, 24.75% fourth-year students.

### 2.2 Tools

The questionnaire includes scales for youth employment anxiety, social media use, and social support. The scales selected for this study are mature scales derived from reviewing domestic and international literature, and their reliability and validity have been tested. All scales use a 5-point Likert scale for scoring.

#### 2.2.1 Youth employment anxiety

The “Vocational Selection Anxiety Scale” compiled by Zhang Yuzhu in 2006 was adopted and based on the use of social media by college students (Zhang and Yao, 2011), a total of 10 terms are obtained by adapting four dimensions: pressure of employment competition, lack of employment support, lack of self-confidence and worry about employment prospects. These items measure the level of anxiety generated by college students using social media, scored from “Strongly Disagree” to “Strongly Agree” on a 5-point scale. Higher scores indicate a higher intensity of anxiety among college students. The coefficient of the scale is *Cronbach’s α* = 0.71.

#### 2.2.2 Social media use

The Social Media Use Intensity Scale adopted the “Facebook Intensity Scale” compiled by Ellison, Steinfield, and Lampe in 2007 (*Cronbach’s α* = 0.83) (Ellison et al., 2007) and the “American College Students’ Facebook Use Scale” adapted by Li Xiao in 2018 from the original scale by Clark N., Lee S. Y., and Boyer L (Gadekar and Krishnatray, 2017). The *Cronbach’s α* coefficient is greater than 0.7. This section contains a total of 12 terms, divided into three factors. The first factor measures time spent on social media, the second factor measures life engagement with social media, and the third factor is emotional retention.

#### 2.2.3 Social support

The “Online and Offline Social Support Scale” compiled by E. Wang and M. Wang in 2013 is adopted. This scale has been validated in the context of Chinese culture (Wang and Wang, 2013). The scale is adapted from the Social Support Scale developed by Leung and Lee, primarily measuring the emotional and informational aspects of social support, as well as the dimensions of positive social interaction and emotional support (Leung and Lee, 2005). This study comprehensively selected 16 items to measure the employment support and online support received by college students. The items are scored on a 5-point scale from “Strongly Disagree” to “Strongly Agree,”” with higher scores indicating better social support conditions for college students. The *Cronbach’s α* coefficient is 0.869.

### 2.3 Data analysis

First, we conducted a bivariate correlation analysis using SPSS 27.0 to examine the relationship between social media use, employment anxiety, and social support. Next, One-Way ANOVA was used to study the differences between gender and grade in employment anxiety Finally (Kim, 2017), Amos 28.0 was used to establish a structural equation modeling (SEM) to examine the mediating effect of social support on the relationship between social media use and employment anxiety (Byrne, 2016). In order to meet the needs of the research, we have revised some existing scales. As suggested by Meyers et al. (2016), we re-examined the reliability and structural validity of the revised scale to ensure acceptable reliability and validity in the context of the study sample. The structural validity of the survey was tested using confirmatory factor analysis (CFA) and SEM in IBM SPSS Amos 28.0 Graphics.

According to the overall model test results of CFA factor analysis, the CMIN/DF value is 1.162, which is less than the adaptation standard of 3 to 5, the RMSEA value is 0.020, which is less than the critical value of 0.08, and the equivalent of statistical tests GFI, NFI, TLI, IFI and CFI are all above the adaptation standard of 0.9. This indicates that the model has a good fit (Kline, 2023). After 5,000 resampling iterations, the hypothesis was tested by calculating 95% confidence intervals.

## 3 Results

### 3.1 Common method bias test

This study employed procedural controls, such as anonymous measurement and reverse-scored items, to mitigate common method bias. Harman’s single-factor test was conducted to assess common method variance. The unrotated exploratory factor analysis extracted 5 factors with eigenvalues greater than 1, and the maximum variance explained by a single factor was 36.50%, which is below the 40% threshold. Therefore, no significant common method variance was detected in this study.

### 3.2 Correlational analysis

Table 1 shows the correlation matrix of college students’ social media use (duration and intensity of use), social support (employment support and online Support) and employment anxiety. The correlation between employment anxiety and usage time is 0.372, the correlation between employment anxiety and use intensity is 0.424, the correlation between employment anxiety and employment support is 0.513, and the correlation between employment anxiety and online support is 0.490, all showing 0.01 level of significance. Therefore, there is a significant positive correlation between employment anxiety and use duration, use intensity, employment support and online support.

**Table 1:** Correlation analysis results.

Item	Usage time	Use intensity	Employment support	Online support	Employment anxiety
Usage time	1				
Use intensity	0.528 * *	1			
Employment support	0.352 * *	0.449 * *	1		
Online support	0.425 * *	0.559 * *	0.440 * *	1	
Employment anxiety	0.372 * *	0.424 * *	0.513 * *	0.490 * *	1

*p < 0.05; **p < 0.01.

### 3.3 Gender differences in employment anxiety, online social support, and social media use

Independent samples t-tests revealed significant gender differences (Table 2). Women scored significantly higher than men on employment anxiety (EA) (M = 3.637, SD = 0.568 vs. M = 2.45, SD = 0.705; *t* = −18.514, *p* < 0.001). Women also scored significantly higher than men on online social support (ONS) (M = 3.344, SD = 0.874 vs. M = 2.798, SD = 0.812; *t* = −6.472, *p* < 0.001), offline social support (OFS) (M = 3.541, SD = 0.888 vs. M = 2.756, SD = 0.917; *t* = −8.702, *p* < 0.001), social media usage intensity (UI) (M = 3.401, SD = 0.884 vs. M = 2.946, SD = 0.851; *t* = −5.245, *p* < 0.001), and usage time (UT) (M = 3.347, SD = 0.862 vs. M = 2.864, SD = 0.83; *t* = −5.706, *p* < 0.001).

**Table 2:** Difference analysis of different gender groups.

Variables	M ± SD		t
	Male (*n* = 198)	Female (*n* = 202)	
Employment anxiety	2.45 ± 0.705	3.637 ± 0.568	−18.514***
Online social support	2.798 ± 0.812	3.344 ± 0.874	−6.472***
Offline social support	2.756 ± 0.917	3.541 ± 0.888	−8.702***
Use intensity	2.946 ± 0.851	3.401 ± 0.884	−5.245***
Usage time	2.864 ± 0.83	3.347 ± 0.862	−5.706***

*p < 0.05, **p < 0.01, ***p < 0.001.

The significant gender differences observed warrant in-depth exploration. Women reporting higher employment anxiety (EA) scores may reflect persistent gender inequalities in China’s labor market, such as gender preferences in certain industries, potential wage gaps, or career advancement barriers. These structural issues may be amplified or more frequently perceived and discussed on social media platforms. Women also reported higher levels of both online (ONS) and offline (OFS) social support, as well as greater social media usage intensity (UI) and time (UT), potentially reflecting gendered socialization patterns. Women typically demonstrate stronger tendencies to seek and provide emotional support, possibly leveraging social media more actively for information acquisition, relationship maintenance, and emotional expression.

Notably, however, higher social support levels did not fully buffer women’s elevated EA (EA correlated strongly with ONS/OFS in women). This suggests that the quality of support, perceived effectiveness of received support, or the nature of specific stressors (e.g., structural gender discrimination) may play critical mediating roles. These findings underscore the necessity of considering deep-seated sociocultural contexts and gender role expectations when addressing college students’ employment anxiety. Interventions must account for systemic inequalities and culturally embedded dynamics that shape anxiety experiences and coping mechanisms.

### 3.4 Structural model

**Figure 2:**
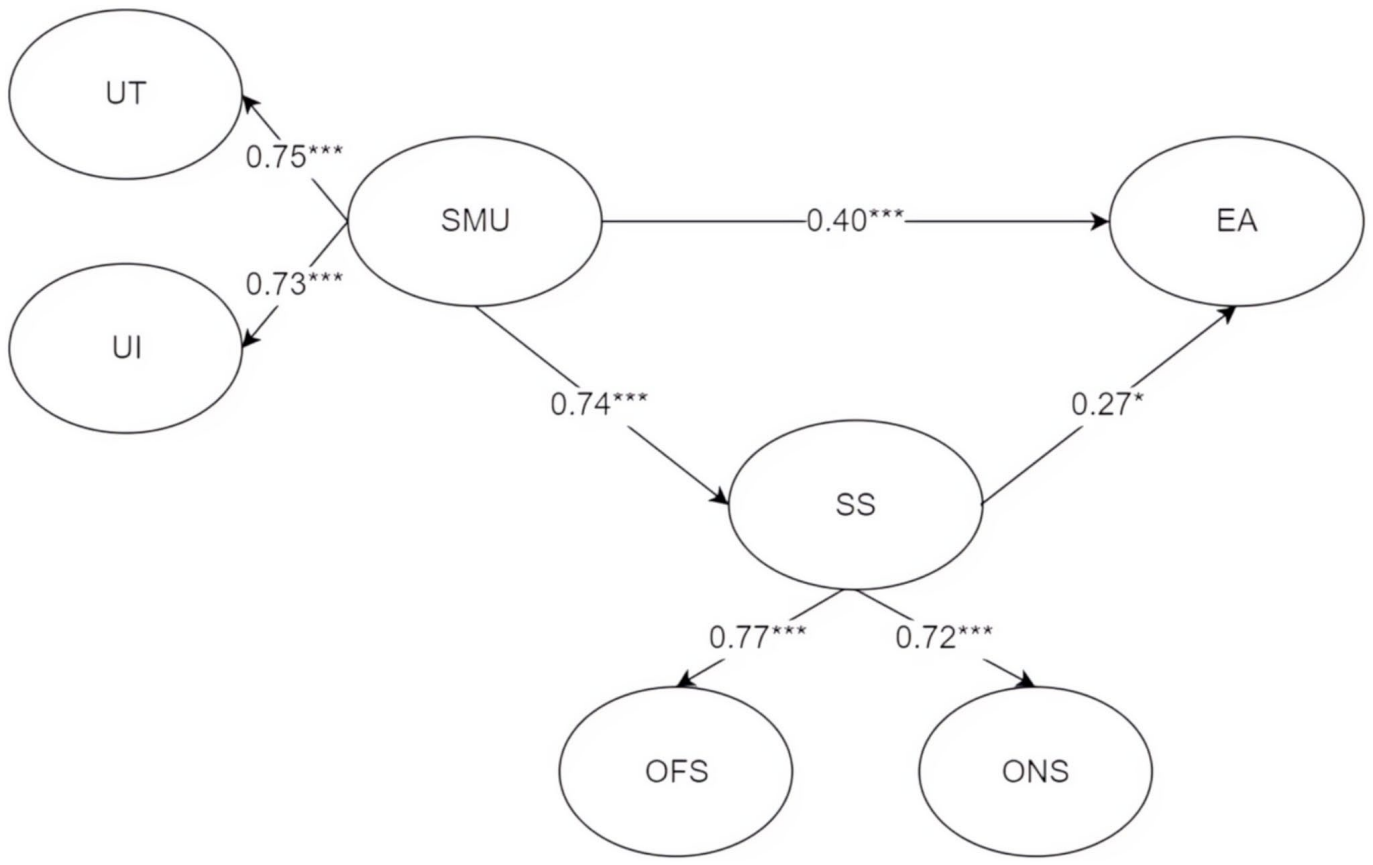
Path analysis diagram and model fit test of social media use, social support, and employment anxiety. SMU, social media use; SS, social support; EA, employment anxiety; UT, usage time; UI, use intensity; OFS, offline social support; ONS, online social support. Each variable in the model was standardized. *p < 0.05, **p < 0.01, ***p < 0.001.

To assess the direct, indirect and overall effects of adolescents’ social media use and social support on employment anxiety, we conducted a path analysis of the established SEM (see Table 3 and Figure 2).

**Table 3:** Fitting indicators for second-order latent variable mediation model.

Fit index	CMIN/DF	RMSEA	GFI	NFI	IFI	TLI	CFI
Fit criteria	<3–5	<0.08	>0.85	>0.9	>0.9	>0.9	>0.9
Result	1.162	0.020	0.907	0.925	0.989	0.988	0.989
Fit Judgment	Fit	Fit	Fit	Fit	Fit	Fit	Fit

As shown in Table 3, from the results of the goodness of fit of the structural equation model, it can be seen that the CMIN/DF value is 1.162, which meets the fitting standard of being less than 3–5. After the model is revised, some fitting indices show that the Root Mean Square Error of Approximation (RMSEA) value is 0.020, which is less than the critical value of 0.08. Moreover, the statistical test measures such as the Goodness-of-Fit Index (GFI), Normed Fit Index (NFI), Incremental Fit Index (IFI), Tucker-Lewis Index (TLI), and Comparative Fit Index (CFI) all meet the fitting standard of being above 0.9. This indicates that this model has a good goodness of fit and an ideal internal quality.

As shown in Figure 2, social media use has significant positive effects on social support (standard path coefficient 0.744, *p* < 0.001), employment anxiety (standard path coefficient 0.405, *p* < 0.001), and intensity of use (standard path coefficient 0.732, *p* < 0.001). The hypothesis is valid. Social support has a significant positive effect on employment anxiety (standard path coefficient of 0.267, *p* = 0.022), online support (standard path coefficient of 0.720, *p* < 0.001), and the hypothesis is valid.

Social media use (SMU) has a significant positive effect on social support (SS) (standard path coefficient 0.744, *p* < 0.001), validating its role as a critical relational tool for college students. This aligns with the theoretical framework, confirming that SMU strongly contributes to perceived social support (both online and offline).

Social support (SS) exhibits a significant positive effect on employment anxiety (EA) (standard path coefficient 0.267, *p* = 0.022), contradicting the expected negative buffering effect. This finding highlights a potential “amplification paradox.” perceived social support may paradoxically intensify anxiety in contexts of heightened employment focus. In China’s sociocultural context, this could reflect implicit pressures from familial and societal expectations for “successful employment,” where perceived support transforms into comparative stressors or heightened responsibility.

Additionally, SMU demonstrates a significant direct positive effect on EA (standard path coefficient 0.405, *p* < 0.001), underscoring the “dark side” of social media use. Even with partial mediation through social support, mechanisms such as social comparison, information overload, and fear of missing out (FoMO) robustly trigger or exacerbate anxiety.

### 3.5 Mediation effect test

After considering gender as a control variable, we adopted a mediation model and Bootstrap method to study the mediating role of social support between social media use and employment anxiety. As can be seen from Tables 4, 5, 95% confidence intervals of total effect, direct effect and indirect effect do not include 0, indicating that social support plays a partial mediating role in social media use and employment anxiety. The mediating effect of “social media use = > social support = > employment anxiety” is established, and the mediating effect value is 0.327, accounting for 64.232% of the total effect.

**Table 4:** Mediating effect analysis.

Item	Symbols	Meaning	Effect size effect	95% CI		Standard error SE value	z value /t value	*p* value	Conclusion
				Lower Limit	Upper limit				
Social media use → Social support → Employment anxiety	a*b	Indirect effect	0.327	0.235	0.358	0.031	10.389	0.000	Partial intermediary
Social media use → Social support	a	X → M	0.631	0.553	0.708	0.040	15.899	0.000	
Social support → Employment anxiety	b	M → Y	0.519	0.407	0.631	0.057	9.100	0.000	
Social media use → Employment anxiety	c’	Direct effect	0.182	0.069	0.295	0.058	3.158	0.002	
Social media use → Employment anxiety	c	Total effect	0.509	0.412	0.606	0.050	10.282	0.000	

**Table 5:** Mediating effect analysis proportion.

Item	Test conclusion	c Total effect	a*b mediating effect	c‘ direct effect	Effect proportion calculation formula	Effect proportion
Social media use → Social support → Employment anxiety	Partial intermediation	0.509	0.327	0.182	a * b / c	64.232%

## 4 Discussion

This study integrates Social Comparison Theory, Stress and Coping Theory, and Social Support Theory to empirically examine the complex relationships among social media use (SMU), social support (SS), and employment anxiety (EA) among college students. The core findings support Hypothesis H1 (SMU positively predicts EA) and reveal a counterintuitive phenomenon with significant theoretical implications: SS acts as a positive mediator rather than a traditional buffering factor between SMU and EA (partial support for H2, but with an opposite directional effect). Additionally, significant gender differences were confirmed, with female students reporting higher levels of EA, SS, and SMU. Below, we discuss the implications, underlying mechanisms, and practical insights of these findings in light of existing literature.

### 4.1 Social media use: an amplifier of employment anxiety

The study confirms a significant direct positive effect of SMU on EA (*β* = 0.405, *p* < 0.001), strongly supporting Hypothesis H1. The core mechanism lies in the inherent features of social media platforms: the constant exposure to others’ job-hunting successes and curated “perfect” career trajectories easily triggers upward social comparisons, leading to relative deprivation and self-doubt (Zubair et al., 2023). Concurrently, information overload and fear of missing out (FoMO) deplete cognitive resources, intensifying uncertainty and anxiety. Passive browsing modes are particularly detrimental, amplifying comparison pressure while weakening genuine social connections. In the Chinese context, platforms like WeChat Moments and Xiaohongshu (Red), characterized by close-knit “familiar social networks” and “curated self-presentation,” may render this comparative pressure more direct and acute.

### 4.2 The “amplification paradox”: the double-edged sword of social support and the reversal of mediation

The study’s core theoretical contribution lies in revealing the “amplification paradox” of social support within the SMU-EA relationship. Contrary to Hypothesis H2 (SS as a buffer), we observed:

SMU significantly and positively predicts SS (*β* = 0.744, *p* < 0.001): This confirms social media as a critical channel for accessing social support (particularly online) for students, validating its connective value.

SS significantly and positively predicts EA (*β* = 0.267, *p* = 0.022): A key reversal. Higher perceived SS correlates with heightened EA.

SS exerts a significant positive partial mediating effect between SMU and EA (indirect effect = 0.327, accounting for 44.672%): SMU not only directly exacerbates EA but also indirectly intensifies it through elevated SS levels.

This “amplification paradox” is not isolated. Zhou and Cheng’s (2022) systematic review highlights the complexity of online social support’s impact on youth anxiety, with some studies showing symptom exacerbation (Zhou and Cheng, 2022). Xie (2023) similarly found that online support amplifies anxiety in high-stress work environments (Xie, 2023), while Sun (2023) observed concurrent increases in SS and anxiety levels through SMU (Sun, 2023).

Why does support amplify anxiety? Drawing on theory and our findings, we propose the following mechanisms:

“Stressful Support” and Implicit Expectations: In China’s high-collectivist culture, social support (especially from families and strong-tie networks) often carries high expectations for “successful employment.” Perceived support may translate into pressure to reciprocate, internalizing rigid success standards. This transforms support from a buffer into a stressor.

Homogenized Support and Escalating Comparisons: On platforms dominated by job-focused peers, support content often homogenizes around shared anxieties. This “group catharsis” or “information sharing” unintentionally reinforces collective perceptions of employment challenges, amplifying their perceived severity and triggering competitive comparisons (She et al., 2023), thereby heightening collective anxiety.

Quality and Efficacy of Online Support: Social media support may involve information overload, superficial encouragement, or competitive content. Its depth, continuity, and emotional authenticity often lag behind high-quality offline support. When perceived as ineffective or shallow, support may induce frustration or self-doubt through upward comparisons (i.e., extended social comparison within the support domain).

Support as a Stress Reminder: Frequent engagement with job-related support may constantly re-activate awareness of the stressful job-search context, hindering psychological detachment and sustaining elevated anxiety.

Thus, the “amplification paradox” reveals how, in high-pressure contexts and collectivist cultures, social support can shift from a resource to a burden. This challenges the simplistic “buffering model” and deepens discussions on the double-edged nature of online social support.

### 4.3 Significant gender differences: a sociocultural lens

The study confirms significant gender disparities. Female students reported higher EA, SS levels, and SMU intensity/duration. This aligns with Farhane-Medina et al. (2022), highlighting women’s heightened vulnerability to employment stress (Farhane-Medina et al., 2022). Integrating China’s sociocultural context.

Employment anxiety differences reflect persistent structural gender inequalities in China’s labor market, such as gender preferences in certain industries, wage gaps, and career advancement “glass ceilings,” which social media may amplify through heightened perceptions and discussions of these systemic inequities, intensifying psychological challenges for female job-seekers. Meanwhile, higher social support-seeking/provision and social media use intensity among women stem from culturally reinforced gendered communication patterns and role expectations, as women are socially encouraged to prioritize emotional expression, relationship maintenance, and social connectivity, driving active engagement with platforms like WeChat and Xiaohongshu for information exchange, emotional venting, and network-building. However, the coexistence of elevated social support and employment anxiety in women underscores the “amplification paradox” within this group, highlighting the dual-edged nature of received support—such as familial expectations embedded in culturally specific stressors—and the need to examine how structural pressures, including workplace discrimination and societal norms, interact with digital behaviors to shape their mental health outcomes.

### 4.4 Limitations

This study acknowledges several limitations that warrant attention. First, the cross-sectional design restricts causal inferences, as temporal relationships between social media use (SMU), social support (SS), and employment anxiety (EA) cannot be established. Future longitudinal research is recommended to validate these dynamics over time. Second, the reliance on self-report questionnaires introduces risks of social desirability bias and response distortion. Combining objective metrics with experimental approaches could enhance validity. Third, while procedural measures mitigated common method variance (CMV), Harman’s single-factor test confirmed no severe CMV (largest factor explained 36.5% of variance, below the 40% threshold). However, residual CMV effects cannot be entirely ruled out, necessitating advanced controls in future work. Additionally, the focus on SMU frequency and online SS overlooks nuanced dimensions, such as platform-specific content or qualitative aspects of support. Exploring variables like psychological resilience or self-efficacy as mediators could refine understanding of EA mechanisms. Finally, the sample’s cultural specificity (Chinese universities) limits generalizability, underscoring the need for cross-cultural comparisons. Addressing these limitations would strengthen the robustness of findings and theoretical implications.

## 5 Conclusion

This study reveals the nuanced interplay between social media use, online social support, and employment anxiety among college students. While high-frequency social media use correlates positively with employment anxiety, online social support paradoxically amplifies this relationship—contrary to its conventional stress-relieving role—likely due to upward social comparisons and pressure from curated online narratives. Gender and grade disparities further highlight the need for context-specific interventions, though these differences warrant deeper exploration. Theoretically, these findings challenge the assumption that online support universally mitigates anxiety, integrating social comparison theory to explain how digital interactions may inadvertently heighten stress. Practically, universities should prioritize digital literacy programs to help students critically evaluate online content, while families and policymakers must foster offline support systems to counterbalance social media’s adverse effects. Programs to prevent SMA must focus on dispositional traits as AATT, social anxiety, respectively unsatisfied need to belong, and promoting the ability to initiate and maintain rewarding social relationships (Stănculescu, 2023). Future research should adopt longitudinal designs to establish causality and explore cultural variations, as well as mediating factors like self-efficacy or resilience, to refine targeted mental health strategies. This study’s cross-sectional design and limited demographic scope underscore the importance of broader, mechanistic investigations to inform scalable interventions for youth career development.

## Data Availability

The raw data supporting the conclusions of this article will be made available by the authors, without undue reservation.
